# A Low-Cost EEG System-Based Hybrid Brain-Computer Interface for Humanoid Robot Navigation and Recognition

**DOI:** 10.1371/journal.pone.0074583

**Published:** 2013-09-04

**Authors:** Bongjae Choi, Sungho Jo

**Affiliations:** Department of Computer Science, Korea Advanced Institute of Science and Technology (KAIST), Yuseong-gu, Daejeon, Republic of Korea; Cuban Neuroscience Center, Cuba

## Abstract

This paper describes a hybrid brain-computer interface (BCI) technique that combines the P300 potential, the steady state visually evoked potential (SSVEP), and event related de-synchronization (ERD) to solve a complicated multi-task problem consisting of humanoid robot navigation and control along with object recognition using a low-cost BCI system. Our approach enables subjects to control the navigation and exploration of a humanoid robot and recognize a desired object among candidates. This study aims to demonstrate the possibility of a hybrid BCI based on a low-cost system for a realistic and complex task. It also shows that the use of a simple image processing technique, combined with BCI, can further aid in making these complex tasks simpler. An experimental scenario is proposed in which a subject remotely controls a humanoid robot in a properly sized maze. The subject sees what the surrogate robot sees through visual feedback and can navigate the surrogate robot. While navigating, the robot encounters objects located in the maze. It then recognizes if the encountered object is of interest to the subject. The subject communicates with the robot through SSVEP and ERD-based BCIs to navigate and explore with the robot, and P300-based BCI to allow the surrogate robot recognize their favorites. Using several evaluation metrics, the performances of five subjects navigating the robot were quite comparable to manual keyboard control. During object recognition mode, favorite objects were successfully selected from two to four choices. Subjects conducted humanoid navigation and recognition tasks as if they embodied the robot. Analysis of the data supports the potential usefulness of the proposed hybrid BCI system for extended applications. This work presents an important implication for the future work that a hybridization of simple BCI protocols provide extended controllability to carry out complicated tasks even with a low-cost system.

## Introduction

Electroencephalogram (EEG)-based BCIs have been of huge interest because of their potential uses. The sensors are noninvasive, therefore, it is relatively much easier to use and the recording procedure is safer. However, it is still the case that the recorded signals are much noisier and less accurate than using invasive techniques. Nevertheless, many interesting studies have demonstrated the feasibility of EEG-based BCI for applications to improve the quality of life of both the physically disabled and healthy people [1]–[14]. Several EEG-based BCI protocols have shown great promise, such as P300 potentials [3]–[5], [9], SSVEP [10]–[12], motor imagery [6]–[8] and ERD/ERS [13]–[14]-based protocols. P300 potential is an approximately 300 ms delayed response to visual stimulus. SSVEP is a response to visual stimulation at a specific frequency. The protocols using P300 or SSVEP are categorized as reactive BCI, which enables users to control an application by detecting indirectly modulated brain signals related to specific external stimuli. Meanwhile, motor imagery or ERD/ERS represent consciously intended brain signals without external events, which is classified as an active BCI protocol. Active BCI is more natural in the sense that thought is directly interpreted as commands. Generally, reactive BCI requires less training and its implementation is relatively simpler than active BCI.

However, it is especially difficult to decode a user’s real intentions from noninvasive brain signals. To simplify the problem, most studies have handled it as a classification problem, which have resulted in a limited number of commands for control. Recently, as an approach to overcome this limitation, the concept of hybrid BCI was developed [15]–[17]. Hybrid BCI, which combines different brain signal types and possibly even including signals not originating from the brain, is appealing because it increase the possibility to provide more varied commands for control. Sequential or parallel processing of different brain signals, with or without external sensing can improve the accuracy of controllability as well as enable extended commands for control. The benefits have been verified by previous studies. Two different protocols of EEG-based BCIs are combined to extend control capacity or accuracy. ERD and SSVEP-based BCIs [18] or sensorimotor rhythm and P300 potential-based BCIs [19] were used together to control two dimensional cursor movement. Similarly, the combined processing of ERD/ERS and SSVEP was investigated for controlling an artificial upper limb [20]. A hybrid BCI using sensorimotor rhythm and P300 was applied to increase the number of commands to control a wheelchair [21]. ERS-based BCI acted as a brain switch to activate or deactivate SSVEP-based BCI operation for orthotics and the hybrid BCI operation yielded a much lower false positive rate than a single conditioned BCI [22]. It is also possible to hybridize EEG-based BCI with a non EEG interface. There are studies that show interfaces which fuse muscular and brain activity [23], while others fused near-infrared spectroscopy (NIRS) and EEG [24]. These systems achieved more accurate and stable results within their respective classification problems. A multi-modal interface consisting of an eye gaze tracker, along with a BCI, resulted in the creation of a robot with an intuitive way for controlling without touch [25]. However, most studies are focused on simple and refined classification problems for evaluation. More complicated tasks should be considered to increase the feasibility of a hybrid BCI for practical applications.

As an example of a complicated BCI task, humanoid robot controls have been selected [7]–[9], [26]. Bell et al [9] applied P300 potentials to target objects which a humanoid robot had to pick up. It is a demonstration that combined a simple BCI with preprogrammed robotic operations. Asynchronous direct control of humanoid robot navigation using sensorimotor rhythm-based BCI was published [7]–[8]. SSVEP-based BCI with a hierarchical adaptive menu was applied to create a simulated robotic arm for household tasks [26]. These papers demonstrated the possibility of noninvasive BCI to control various motions of humanoids. Even though the published results have been impressive, more-detailed multi-task actions by EEG-based BCI still needs to be explored. When a bigger variety of performable tasks are available, the humanoid robot can better work as a surrogate of the user. More natural control of the surrogate becomes possible when the user thinks it is a replacement of his or her body. The use of invasive or fMRI-based BCIs attained more detailed control of a humanoid [27]–[28]. However, the increased size and cost of these invasive recording devices, along with the stipulations placed on the users, makes these devices less attractive for general use.

This work investigates a noninvasive hybrid BCI paradigm for the actuation of, and the recognition of objects by, a humanoid robot, which is applicable to real-life scenarios. In addition, this work intentionally uses a low cost BCI system that is more convenient for users and more portable. In addition, simple BCI protocols are selected for their ease of adaptability to different users. The simplicity of the system increases its applicability to other real-world applications. Therefore, this work aims to overcome the limitations of relatively low-quality data from a low-cost system by suggesting algorithmic solutions. This work evaluates whether a hybrid BCI technique can be a solution to improve the reliability and strengthen the controllability of a low cost BCI system. The objective of this work is to demonstrate that it is possible to use a low-cost hybrid BCI system to perform multiple tasks with a humanoid robot while giving the user the illusion of embodying the robot.

## Methods

### Scenario description

**Figure 1:**
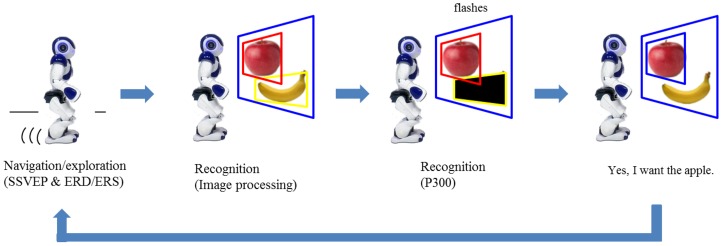
Overall scenario of humanoid navigation/exploration and recognition tasks.

Figure 1 illustrates the overall scenario. The scenario consists of humanoid navigation/exploration and recognition tasks through EEG-based BCIs. The humanoid surrogate, operated through a combination of SSVEP and ERD-based BCIs, navigates and explores a maze. When it encounters any objects, represented by photos of various fruits randomly scattered throughout the maze, the objects are detected by a simple image processing technique. The surrogate robot recognizes whether the detected object is the object which the user is looking through the P300 potential-based BCI.

**Figure 2:**
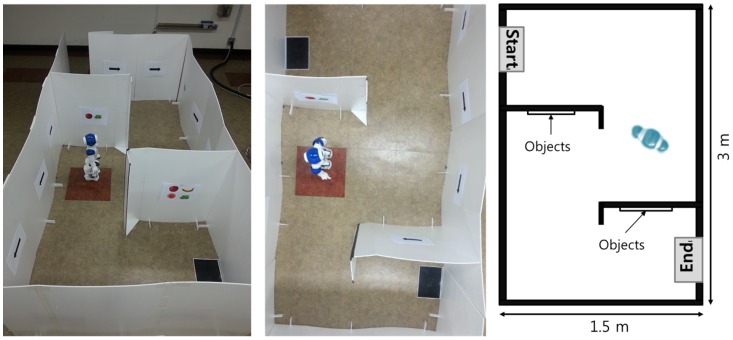
Experimental setting.

The surrogate robot was placed at the starting position in the maze. Figure 2 shows the experimental setting of the 1.5×3 m^2^ maze. At several corners, several objects were placed. A subject had to navigate the surrogate robot through the pathway relying on visual feedback. Whenever each object was within view, the surrogate robot recognized whether it was a favorite object of the subject or not through the signals recorded from the subject’s brain.

### Subjects and EEG data acquisition

Among the candidates that volunteered, inclusion and exclusion selection criteria were used to ensure a homogeneous population for the purposes of the study’s conclusions. The inclusion criteria were 1) users within the 20–30 year age range; 2) users within the same gender group; 3) users of the same laterality (all right handed). The exclusion criteria were 1) users with a history of central nervous system abnormalities; 2) users taking any psychiatric medications; 3) users with epilepsy, dyslexia, or experiencing hallucinations; 4) users with any previous experience with BCI. According to the criteria, five healthy male volunteer subjects (age 23.4±3.8 years) participated in the experiment. All subjects gave written informed consent. The KAIST Institutional Review Board approved the proposed experimental protocol of this study.

To validate the application of a low-cost BCI system for feasible task performance, brain signals were recorded using an Emotiv Epoc headset (Emotiv Systems Inc., USA) [29]. The Emotiv Epoc BCI recording system is much cheaper than state-of-the-art BCI recording systems. The headset’s ease of usage, portability and simplicity of operation make it an attractive headset to use within this study. It has been tested in previous studies [3], [30]–[32]. The headset can measure brain activity through a total of fourteen electrode channels around the sensorimotor cortex. Using the headset, data was collected at a sampling frequency of 128 Hz from the fourteen channel layout; AF3, AF4, F3, F4, F7, F8, FC5, FC6, P7, P8, T7, T8, O1, and O2 with respect to the 10–20 system.

### Design of navigation and exploration mode

**Figure 3:**
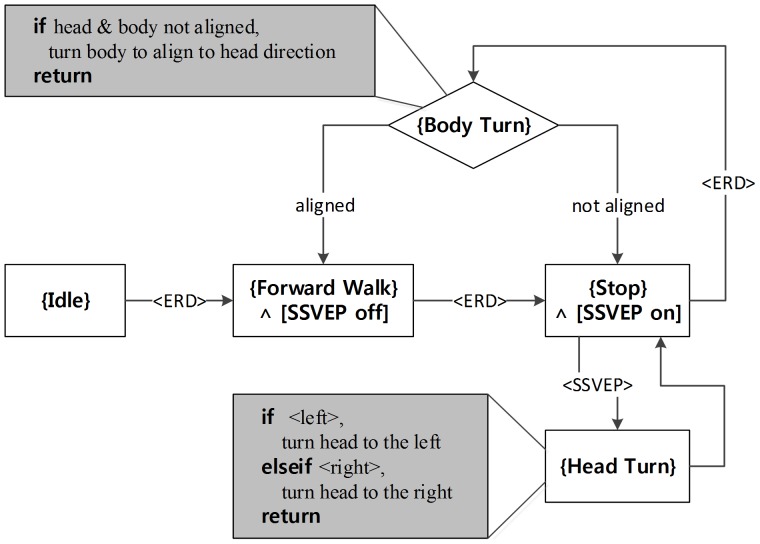
Proposed algorithm for navigation and exploration mode.

An EEG-based BCI was used for the humanoid motion control. To perform the navigation and exploration of the humanoid, the EEG-based BCI protocols, SSVEP-based and ERD-based, were combined. In this work, navigation consists of two behaviors, forward walk and body turn (left or right) while exploration is made up of turning the head (left or right). The navigation mode enables the humanoid to move around in the maze. During navigation and exploration, a user can obtain visual feedback about the environment. Figure 3 illustrates the organization of the overall algorithm used to implement navigation and exploration.

To minimize burden on the subject and promote a more convenient interface, a hybrid strategy was proposed. Initially, whenever the ERD-based BCI detected a user-specified motor imagery, the humanoid changed its state as indicated by “<ERD>” in Figure 3. It is similar to a “state transition switch”. Foot motor imagery was recommended, but not mandated. During the experiments, each subject was allowed to choose his or her favorite motor imagery such as foot or hand motor imageries, which was well classified. While no specific imagery was detected, the humanoid robot maintained its state. Therefore, the user did not have to continuously focus on their specified imagery, which minimized the effort needed. The reactive SSVEP-based BCI requires displaying flickering stimuli. This flickering could distract a user when he or she did not wish to use the SSVEP-based BCI [22]. Thus, SSVEP-based BCI was turned on as needed. Once SSVEP-based BCI was activated, head turns could be actuated by responding to the flickering stimuli. In this study, <left> and <right> states were discriminated by SSVEP-based BCI using two visual stimuli flickering at their respective specific frequencies. The humanoid rotated its head in the selected direction by 3 degrees per decision. By appropriately turning the head, a user could explore the environment from visual feedback. Turning the humanoid body to either the left or right was implemented by turning its head toward the desired angle through SSVEP-based BCI and then operating a state transition through ERD-based BCI. When its head and body were not aligned, that is, the angle between head and body was greater than 9°, an ERD switch provoked the body to turn to align itself in the same direction as the head. The humanoid did not respond to any other commands from any of the BCI protocols during alignment. The alignment behavior resulted in the body turning to the desired direction. Once the body was aligned, the humanoid remained still and waited for the next command. On the other hand, if head and body were aligned when ERD-based BCI operated, the humanoid simply walked forward without aligning the body first.

The proposed algorithm in Figure 3 was designed by combining simple BCI protocols with a postural dependent control scheme to achieve desired performance with less effort and more comfort. That is, an identical classification outcome can implement different robot behaviors depending on the robot postures.

### Design of recognition mode

**Figure 4:**
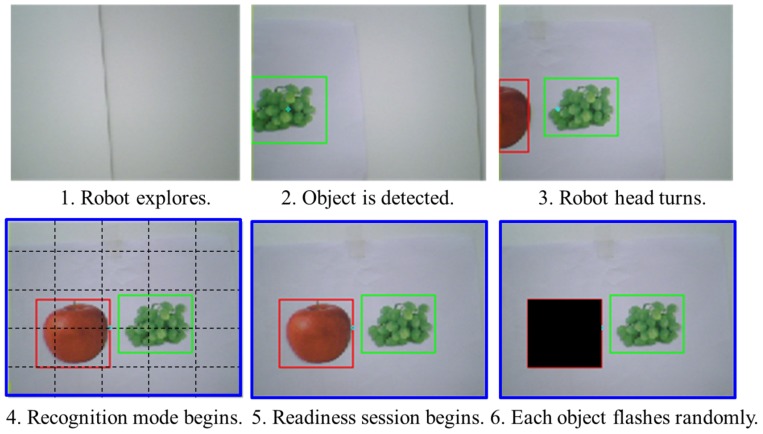
Proposed procedure of recognition mode.

**Figure 5:**
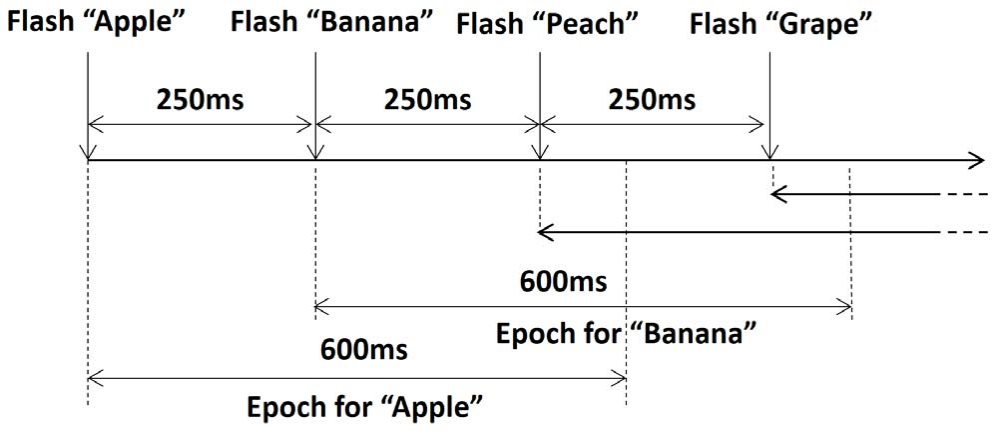
Timing diagram of stimulus events and corresponding epoch windows for EEG signals.

When objects, which were fruits such as apples and bananas in this study, appeared in the view of the humanoid surrogate, a very simple color filtering technique detected them as shown in Figure 4. Color information of each object in the white background was used to detect the object’s region. Then, a rectangular box appeared to indicate the region. Each subject turned the robot to position the center of the box near the center of the surrogate robot’s view. When multiple objects were detected, each subject tried to locate a virtual center of all of the rectangular boxes near the center of the view window. The virtual center of multiple objects was a center of gravity of objects. When the height and width of the whole view window are divided into five zones, the middle zone indicates the central region of the view window. When the virtual center of rectangular boxes was located within the central region, the recognition mode began. Subjects were informed of the start of recognition mode by turning the view window’s border blue. The humanoid remained still during recognition mode. Once the recognition mode turned on, a 4 s rest was provided before each object region started flashing for P300-based BCI operation. The rest aimed to provide subjects with a short break to be ready for P300 operation. Once P300-based BCI began, flashes occurred every 250 ms in a random order. While an object region flashed, the other object regions were blacked out. An epoch of EEG signals corresponding to the flashing of a particular object is a 600 ms window of time from when the flashing of an object starts. Figure 5 illustrates the course of time of the flash stimulus and corresponding epochs in EEG signals. Each epoch was checked for P300 potential. If P300 potential was detected, the surrogate robot interpreted that the corresponding flashed object was a user's favorite, otherwise it was not a favorite object of the user. During the real-time humanoid experiments, the results of the object recognition were displayed on the monitor for 2 s before transitioning back to navigation/exploration mode. The blue border disappeared once the recognition mode was over.

### Designs of BCI protocols

The proposed hybrid BCI consisted of three BCI protocols. Each of the protocols relied on a simple but effective algorithm in order to carry out a two-class classification with a low-cost recording system. In each case a simple algorithm was selected which could solve its two-class classification problem with high accuracy while requiring little training.

#### ERD-based BCI protocol

According to Figure 3, the humanoid changed states if pre-designated motor imagery was detected. Common Spatial Patterns (CSP) algorithm [33], a popular technique in EEG-based BCI, was used to extract features to identify the specified motor imagery. EEG signals were filtered between 8 and 30 Hz, which mainly contain motor imagery information covering the mu and beta frequency bands. Let 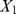 represent a set of EEG signals corresponding to the specified motor imagery, and 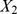 represent the remaining EEG signals. 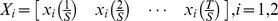



where 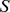 is the sampling rate, and 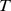 is the number of samples. The dimensionality of each vector 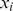 is equivalent to the number of electrode channels.

CSP finds spatial filters **^W^** D_Dd_________ the following function. 
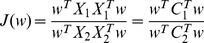



Each 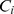 indicates the spatial covariance matrix of an associated class assuming a zero mean for EEG signals. The zero mean assumption is met by preprocessing the EEG signals through a band-pass filter. Using the Lagrange multiplier method, the optimization problem is transformed to be a standard eigenvalue problem. Therefore, the spatial filters **^F^** 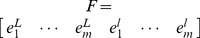


where 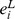 and 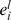
are the ith largest and lowest principal directional eigenvectors of 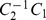 respectively. 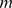 was set to be 2 in this study.

Then, features 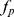, 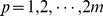 **^,^** are assigned to be
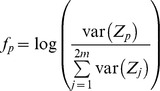



where 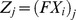 for either active or idle states.

Finally a classifier to discriminate the two classes was constructed by applying the Support Vector Machines (SVM) algorithm [34] with a linear kernel to the features. SVM was selected because of its good generalization properties [35]. The MATLAB functions for SVM computation were used in this implementation.

#### SSVEP-based BCI protocol

SSVEP-based two-class classification, which indicates left and right head turns, was designed based on Canonical Correlation Analysis (CCA) [36]. CCA works on two sets of variables. A set of variables are EEG signals 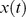 recorded from several channels of the headset. The other set describes stimulus signals 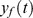 which are set at a particular frequency 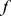 as follows.
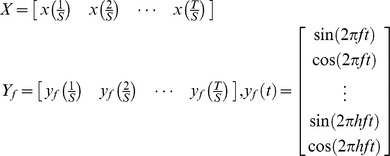



Where 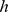 is the number of harmonics. In this study, 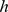 was set to be 1.

Two sets of linear combinations, called canonical variants, are defined to be 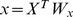 and 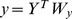. CCA finds the weight vectors 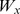 and 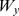 that maximizes the correlation between 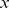 and 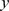:



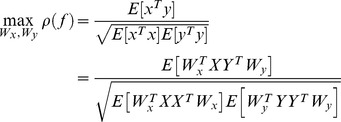
 (1)

Two stimulus frequencies 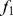 and 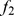 were designated to indicate left and right turn states respectively. EEG signals were recorded responding to flickering at the two frequencies. When correlation maximizations were conducted for the two stimuli, the left turn state was selected by checking the correlation values, 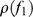 and 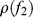. This is explained in detail in the following section. This enables the exploration of the environment by turning the head, while turning of the body can also be implemented by then aligning the direction of the humanoid body to that of the head.

The two channels O1, and O2 of the headset were used for SSVEP-based BCI protocol because they cover the occipital area where SSVEP are detected well. Collected EEG signals from the two channels were filtered between 4 and 50 Hz to reduce artifacts using a fifth order Butterworth band-pass filter.

#### Selection of ERD and SSVEP-based BCI protocols

**Figure 6:**
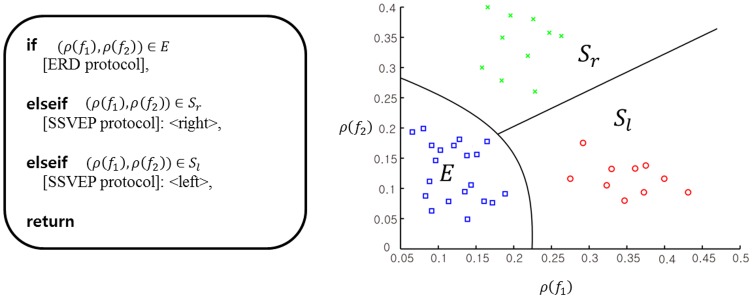
Proposed selection rule of ERD and SSVEP-based BCI protocols.

As EEG data was recorded in real-time, a selection between the two BCI protocols was required. Using training data of the left and right turns in SSVEP as well as that of ERD, 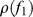 and 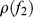 were computed from equation (1). Then, the distribution of 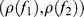 was plotted as shown in Figure 6. SVM set a quadratic classifier to decide which BCIs protocol to select. ERD-based protocol operated when the recorded data point was located in the “*E*” region, otherwise the SSVEP-based protocol ran. When the SSVEP-based protocol was on, the discrimination between the left and right head turns, the “*S_r_*” and “*S_l_*” regions in Figure 6, was identified by a linear classifier obtained through SVM.

#### P300-based BCI protocol

This work implemented a simple P300-based BCI protocol using the xDAWN spatial filter which was proposed to enhance evoked potentials [37]. The method improves the quality of the evoked responses by taking into account both the signal and the noise, hence it is appropriate to be applied in our case, which uses a low-cost recording system. Collected data was projected on to a three-dimensional signal subspace constructed by the spatial filter. Then, a Bayesian linear discriminant analysis classifier [38] was applied to identify the evoked P300 potentials. In the protocol, each epoch was recorded after the stimulus onset. They were band-pass filtered between 1 Hz and 20 Hz and down-sampled from 128 Hz to 32 Hz.

### Dynamic fading feedback rule

In order to achieve robust classification results with the proposed BCI system and guard against misclassification results, this work adapted the dynamic fading feedback rule proposed in [7]. The principle of the rule is to confirm a command based on consecutive classifications by setting a selection level to avoid abrupt false classification. The rule is as follows: The selection level is initially set to zero and the first classification results in a command candidate. Whenever further classifications result in an identical command candidate, the selection level increases by one. Otherwise the selection level decreases by one. If the selection level reaches zero, the next classification assigns new classification candidate. Whenever the selection level reaches four, the command candidate is finally confirmed and the command is sent to the surrogate robot. In this paper’s implementation, we applied this rules for hybridization of SSVEP-based and ERD-based protocol. Each classification was determined every 250 ms.

### Interface system

**Figure 7:**
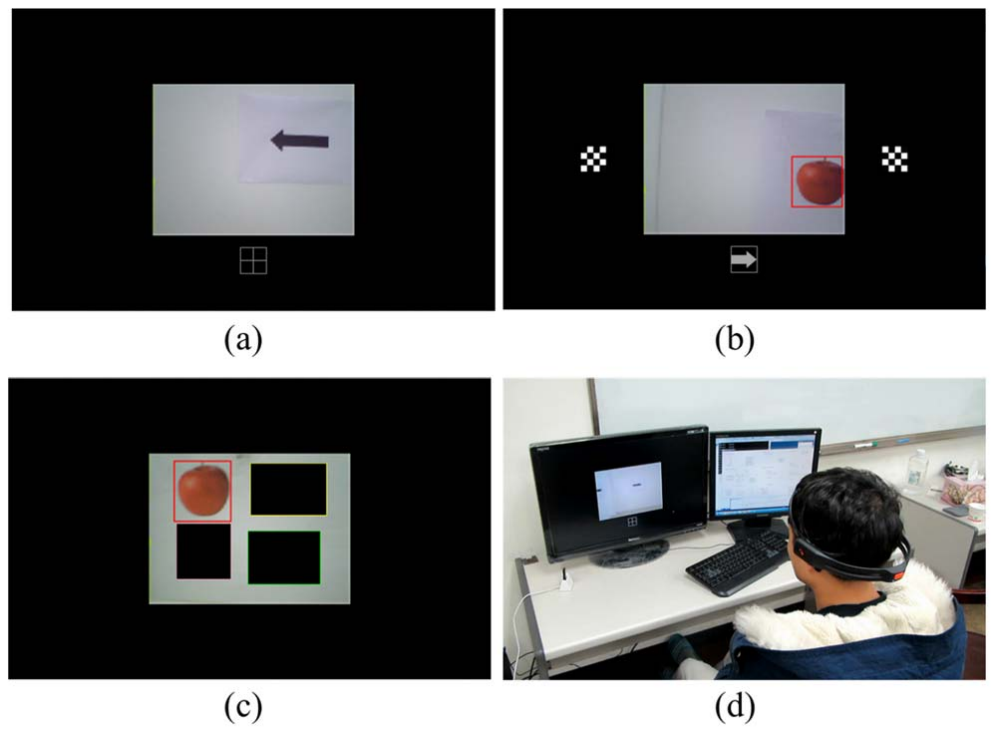
The design of the display during (a) walking straight, (b) turning head, and (c) recognition. (d) The interface being used by a subject.

The subject sat comfortably looking at a monitor wearing the recording headset. A video stream from the robot’s camera was displayed in the center of the screen at 10 fps. Any command sent to the robot by way of interpreting brain signals was indicated below the view window (see Figure 7(a)). When the SSVEP protocol was active, flickering rectangles at the stimulus frequencies 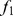 and 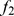 were located on the left and right sides of the video stream (see Figure 7(b)). During object recognition, neither the sent command nor SSVEP flickering rectangles were visible while the boxes surrounding the objects flashed randomly in the camera view (see Figure 7(c)). Figure 7(d) shows a subject interacting with the robot through the described interface.

### Overall system description

**Figure 8:**
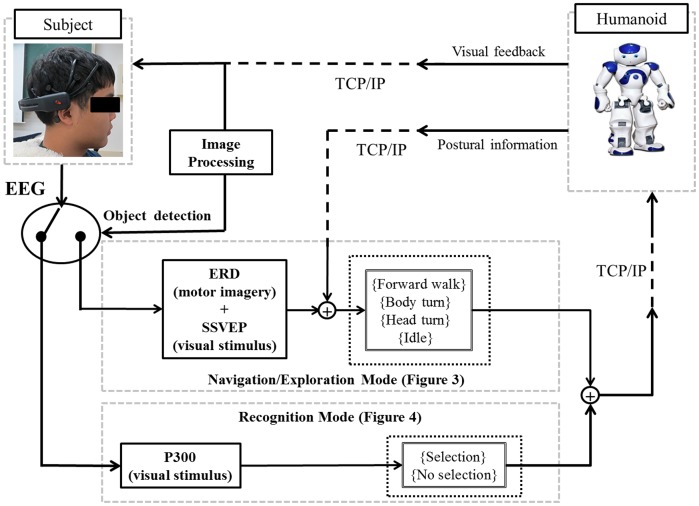
The system architecture.

The surrogate robot used in this work was a Nao humanoid robot (Aldebran Inc., France) with 25 degrees of freedom. The robot is equipped with monocular vision on its head that can provide a front view to the user. The robot was configured to walk at a speed of 3.3 cm/s and make turns at a speed of 0.13 rad/s. These speeds were selected to ensure stable robot movement. A subject sat comfortably in front of a computer while the robot was separately located in the maze. A TCP/IP protocol transferred data wirelessly between the interface computer and the robot. Figure 8 illustrates the overall system.

## Experimental Results

### Real-time humanoid navigation/exploration experiment

Each subject was first asked to complete a real-time humanoid navigation/exploration task without the object recognition task. To decide the classifiers for the ERD-based and SSVEP-based BCI protocols respectively, the following procedure was followed. For the ERD-based protocol, EEG signals were recorded while each subject remained neutral for 5 s and imagined a specific motor imagery for 5 s. Each subject selected a specific motor imagery, such as moving a right hand, which they could easily imagine. Each subject repeated this 20 times. Then, a dataset per 2 s time window with 250 ms increments was obtained. The method explained in the ERD-based BCI protocol section was applied to a collection of datasets to obtain a classifier. A tenfold cross-validation was assessed to evaluate the classification performance. The specific stimulus frequencies for the SSVEP-based protocol were selected to be 12 and 15 Hz for all subjects through empirical pre-tests. Evoked response data was manually analyzed to find distinguished frequency peaks by taking into account the specification of the low-cost recording headset. In a previous study [32], the limitation of stimulus frequency up to 15 Hz was discussed using the selected recording headset. Once two stimulus frequencies were decided, data was acquired using flickering checkerboards at the designated frequencies. Each subject was asked to look at flickering checkerboards for 5 s; this was repeated a total of 20 times. Then, again, a dataset per 2 s time window with 250 ms increment was obtained. The method explained in the SSVEP-based BCI protocol section was applied to this collection of datasets to obtain a classifier. Again, a tenfold cross-validation was used. When adequate cross-validation accuracies were obtained, the estimated classifiers were used for completing the real-time task. Table 1 indicates the validation results as well as the information transfer rate (ITR).

**Table 1:** Cross-validation accuracies and ITRs of BCI protocols used for navigation/exploration.

Subject	A	B	C	D	E	Overall
ERD cross-validation accuracy (%)	76.8	86.7	90.1	81.8	87.5	84.6 (±5.3)
ERD ITR (bits/min)	6.6	13	16.0	9.5	13.7	11.8 (±3.7)
SSVEP cross-validation accuracy (%)	92.2	80.8	84.9	79.5	84.6	84.4 (±5.0)
SSVEP ITR (bits/min)	18.1	8.8	11.6	8.1	11.4	11.6 (±3.9)

ERD and SSVEP-based protocols achieved an overall accuracy of 84.6 and 84.4%, respectively. Both protocols also resulted above 11 bits/min ITR. The ERD cross-validation accuracy was the worst for subject A at 76.8% while SSVEP cross-validation accuracy was the worst for Subject D at 79.5%.

**Figure 9:**
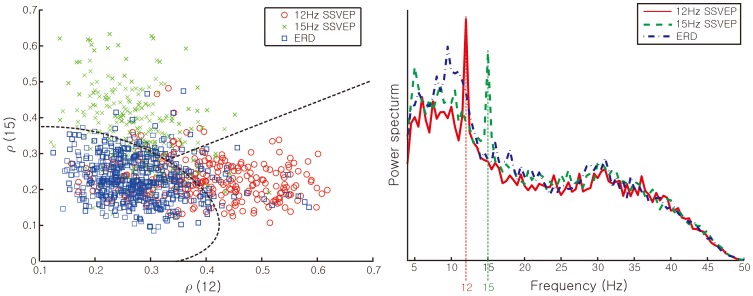
SSVEP and ERD-based BCI selection: (a) selection classification region: dotted lines indicate decision boundaries, and (b) average brain activities specified from O1 during BCI operation of subject C.

With confirmed classifiers of the SSVEP and ERD-based protocols per subject, the classifier for selection between SSVEP and ERD-based protocols was assigned. The assigned classifier was also tested before the real-time experiments. Average accuracy over all of the subjects was 73.0 (±3.6)%. The worst performer was subject B, whose accuracy was 68.8% while subject C achieved the highest accuracy of 78.3%. Figure 9(a) illustrates an example of the classified region obtained based on data samples from a particular subject. Figure 9(b) exemplifies that SSVEP and ERD brain activities are classified according to the selection rule between SSVEP and ERD-based BCIs. Due to the limited specifications of the low-cost headset, stimulus frequencies of 12 and 15 Hz, had to be selected for SSVEP. Meanwhile, motor imagery signals for the ERD-based BCI tended to peak around the mu band (8∼13 Hz). The peak of motor imagery signals overlapped with that of SSVEP stimulus frequencies and therefore could be confused.

Real-time humanoid navigation/exploration experiments were conducted in the environment described in the scenario description section. To evaluate the experiment’s performance, several metrics for each task were collected as done in a previous study [7]: total time taken during task, total travelled distance during task, total number of steps taken during forward movement, total number of steps taken during body turn, summation of the angles the head turned during exploration, total number of transitions between navigation and exploration modes, and total number of collisions with the wall. Furthermore, the BCI-based performance was compared with manual keyboard control-based performance. Table 2 summarizes the overall performance of the real-time navigation task as carried out by the five subjects with respect to the selected performance metrics. The performance metric values were averaged across three trials for both BCI and manual control cases.

**Table 2:** Real-time humanoid navigation/exploration control results.

Subject	Session	Total time (sec)	Distance travelled (cm)	Forward steps (times)	Turning steps (times)	Explored angle (rad)	Transitions (times)	Collisions (times)
A	BCI	507.8	434.4	134.0	44.7	7.0	26.0	0.3
	Manual	483.4	468.0	152.3	45.3	6.8	17.3	0.3
	BCI/Manual	1.05	0.93	0.88	0.97	1.02	1.50	1.00
B	BCI	542.9	432.5	141.7	42.0	7.1	19.7	0.3
	Manual	416.9	440.7	137.3	41.3	6.6	16.3	0.7
	BCI/Manual	1.30	0.98	1.03	1.02	1.09	1.20	0.50
C	BCI	582.8	461.0	150.0	46.7	8.2	19.7	0.3
	Manual	477.9	485.9	143.0	48.3	7.3	19.3	0.3
	BCI/Manual	1.22	0.95	1.05	0.96	1.12	1.02	1.00
D	BCI	609.6	462.8	145.3	41.3	9.1	29.0	0.3
	Manual	460.9	467.7	149.3	41.3	7.3	20.7	0.7
	BCI/Manual	1.32	0.99	0.97	1.00	1.25	1.40	0.50
E	BCI	570.2	427.4	136.3	36.7	7.9	18.7	0.3
	Manual	478.4	410.1	131.7	39.3	6.7	18.0	0.3
	BCI/Manual	1.19	1.04	1.04	0.93	1.19	1.04	1.00
Overall	BCI (±SD)	562.7 (±59.0)	443.6 (±20.6)	141.5 (±7.2)	42.3 (±4.5)	7.9 (±1.0)	22.6 (±5.1)	0.3 (±0.5)
	Manual (±SD)	463.5 (±43.6)	454.5 (±32.7)	142.7 (±10.4)	43.3 (±5.0)	6.9 (±0.7)	18.3 (±3.2)	0.5 (±0.5)
	BCI/Manual (±SD)	1.22 (±0.11)	0.98 (±0.04)	0.99 (±0.07)	0.98 (±0.03 )	1.13 (±0.09)	1.23 (±0.22)	0.80 (±0.27)

Working under the assumption that the manual control performance was nominal, the BCI-based performance was compared with the manual control performance by computing the metric ratios as summarized in Table 2. All subjects took more time to complete the experiment using the BCI-based approach than manual control. The average ratio of BCI-controlled execution time to manual-control execution time was 1.22. The ratio is a little less than the value (1.35) obtained using sensorimotor rhythm-based BCI control under a similar experiment scenario [7]. Because the total time includes the robot operation, BCI and manual control execution cannot be compared only with the time metric. As for the distance travelled, with the exception of subject E, all subjects were able to attain a ratio of less than 1. However, all of the ratios are close to one, which indicates the same distance was travelled. With respect to the numbers of forward steps and turning steps, BCI and manual control performed quite similarly, considering their ratios. With these results, BCI-controlled navigation task performances were comparable with manual-based performances. Looking at the accumulated angle of the head, which represents exploration, its ratio was greater than one for all subjects, having an average of 1.13. Although not significantly different, the interpretation is that BCI-based controls resulted in more exploration. BCI control tended to transition between navigation and exploration modes about four times more than manual control, which resulted in an average ratio of 1.23. In most cases, subjects collided with the wall only once during a particular run of the maze, regardless of whether BCI or manual controls were used. Overall, the humanoid behaviors as executed by the subjects are comparable when considering BCI control and manual control.

### Humanoid object recognition experiment

**Figure 10:**
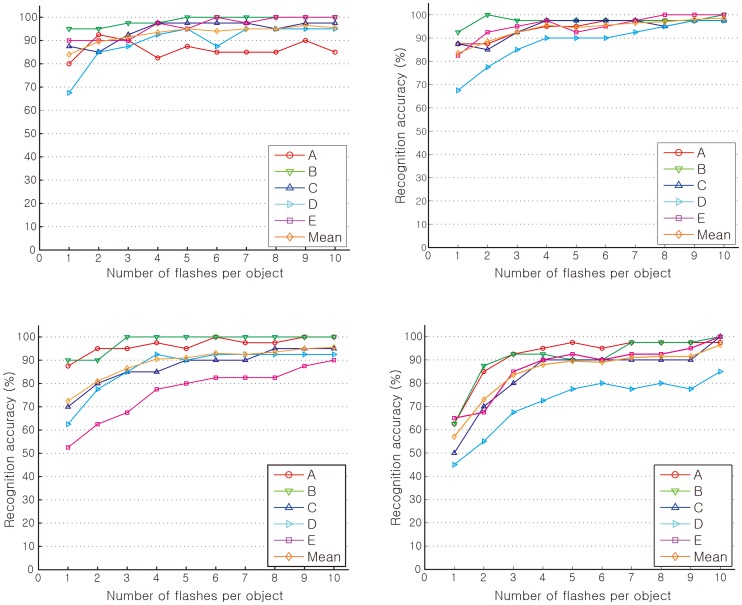
Per subject recognition accuracies over the number of flashes per object of (a) two objects on the computer monitor, (b) four objects on the computer monitor, (c) two objects through robot vision in real-time, and (d) four objects through robot vision in real-time.

To complete the real-time object recognition task, the five subjects used a P300-based BCI to conduct object recognition. To identify classifiers, EEG data was first collected beforehand by having each subject recognize four objects on a computer monitor. In each trial, each object was flashed up to 10 times in a randomly selected order. This process was repeated for a total of 40 trials. EEG data obtained during the trials was used to calculate a classifier using the method mentioned in the selection of ERD and SSVEP-based BCI protocols section. Then, tests were performed for four cases: recognition between two and four objects on the monitor, and recognition between two and four objects by viewing them through the robot’s camera. Each case was also performed 40 times. During the robot vision test, the surrogate robot stood about 35 cm in front of the prearranged objects. Each subject performed the object recognition task looking at the video stream sent from the robot. The image view outlined the objects in real-time. Figure 10 illustrates the test results. Overall, the accuracy of recognition increased as the number of flashes increased. Subjects generally performed better with tests performed with the monitor alone than through viewing objects through the robot's camera. Even so, recognizing objects through the robot's camera still attained around 90% accuracy with all but one of the subjects when flashing each object five times. Flashing each object 5 times, the average recognition accuracy was 91% for two objects and 89.5% for four objects when objects were detected through the robot's camera. Based on these results, it was decided that five sequential flashes would be used during real-time humanoid experiments**.** Therefore, it is expected that object recognition through the P300 would take 2.85 s for two objects and 5.35 s for four objects.

The ITR of the P300-bsed BCI is summarized in Table 3. An ITR of approximately 12–18 bits/min was attained. Specifically, real-time robot vision-based recognition resulted in an ITR 12–15 bits/min. However, for each of two and four object recognition scenarios conducted through the robot’s camera, one particular subject’s inferior performance lowered the average ITR. In every case the highest ITR was over 20bits/min.

**Table 3:** ITRs during various object recognition scenarios.

Subject	Two objects on monitor	Four objects on monitor	Two objects through robot vision	Four objects through robot vision
A	9.6	18.3	15.0	20.1
B	21.1	20.1	21.1	15.4
C	17.5	20.1	11.2	15.4
D	15.0	15.4	11.2	9.8
E	15.0	16.8	5.9	16.8
Overall	15.6 (±4.2)	18.1 (±2.1)	12.9 (±5.6 )	15.1 (±3.7)

### Real-time humanoid navigation/exploration and recognition

After performing each task of navigation/exploration and object recognition separately, the real-time humanoid navigation/exploration and recognition task was conducted by the five subjects with the previously discussed set-up for the different modes. Each subject repeated the task three times using the classifications determined in the two previous sections. While traversing through the maze, recognition tasks were requested to be performed two times as shown in Figure 2. Fruit images were randomly selected among five different varieties. In each object recognition task, two to four objects were arbitrarily displayed. Table 4 summarizes the overall performance results obtained from averaging over the three trials.

**Table 4:** Real-time humanoid navigation/exploration and recognition control results.

Subject	Total time (sec)	Navigation/exploration time (sec)	Recognition time (sec)	Distance travelled (cm)	Forward steps (times)	Turning steps (times)	Explored angle (rad)	Transitions (times)	Collisions (times)	Object recognition success rate
A	682.0	661.3	20.7	456.9	149.3	78.0	13.8	33.3	0.3	6/6
B	746.1	722.4	23.7	472.9	163.3	70.0	13.8	25.0	0.0	6/6
C	757.1	725.4	31.6	483.9	149.3	61.3	12.9	27.7	0.7	6/6
D	807.9	774.6	33.3	503.8	164.7	81.7	13.7	32.7	0.3	5/6
E	954.2	927.4	26.8	526.9	162.3	78.3	13.6	36.0	0.0	4/6
Overall	789.5 (±116.8)	762.3 (±116.0)	27.2 (±5.7)	488.9 (±39.7)	157.8 (±11.4)	73.9 (±13.0)	13.6 (±1.2)	30.9 (±6.8)	0.3 (±0.5)	5.4/6

As seen in Table 4, most of the time during the trial was spent in navigation/exploration mode, 762.3 s on average. The cumulative times for navigation/exploration are greater than those in Table 2 due to more time being spent positioning the robot near objects. Also, the total travelled distances increased compared with those in Table 2. It can be clearly understood that the robot had to navigate to the locations of objects, rather than take the shortest path to the finish line, as was the case with the navigation/exploration-only task. Therefore, forward steps, turning steps, explored angles, and transitions were generally greater than those in Table 2. The favorite objects of subjects were well recognized in most cases. Recognition time is the average amount of time spent in recognition mode. Specifically, it is the average amount of time between entering recognition mode and returning to navigation mode. On average, the entire object recognition task took a total of 27.2 s. This implies that the object recognition itself took about 13.6 s. The recognition time includes the 4 sec readiness interval, the 2 s display of the results, the robot launching interval as well as the P300-based BCI operation. Using the dynamic feedback rule, it took at least 1 s for the robot to start moving again.

**Figure 11:**
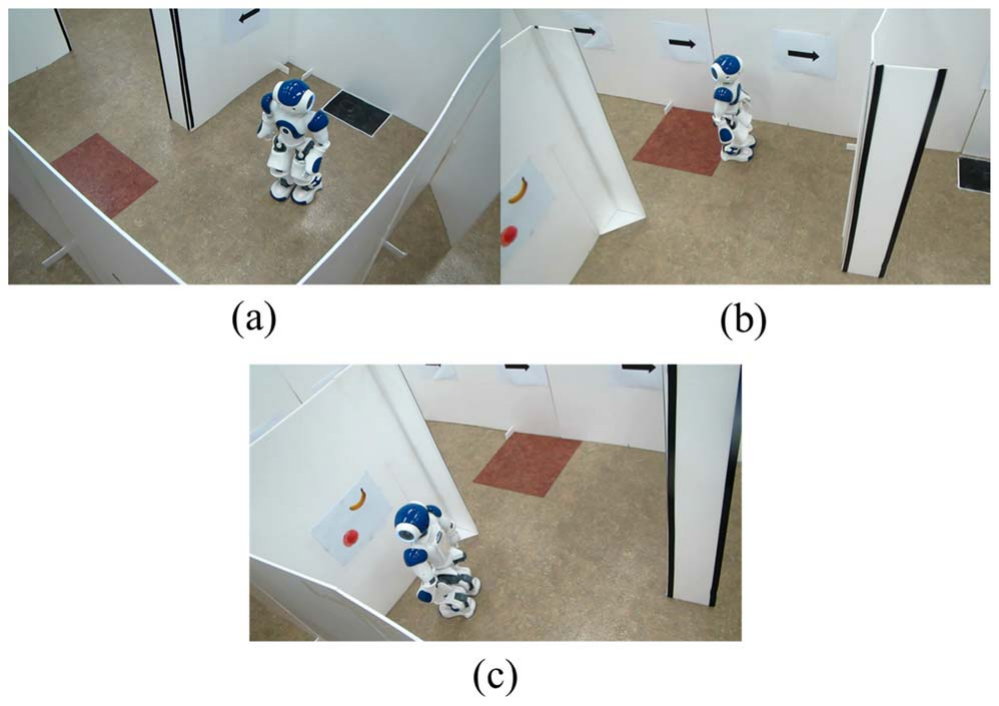
Pictures taken during a real-time humanoid experiment: (a) walking forward, (b) looking around, and (c) recognizing an object. A movie clip demonstrating the experimental performance has been included as Video S1.

Figure 11 shows some pictures taken during a trial being performed by a subject. In the maze, the surrogate robot navigated, gathered information about its environment, and looked at fruit images on the wall for object recognition while reaching a destination from a starting point. A video of the experiment in action has been included as Video S1.

## Discussion

This study shows that a hybridization of simple BCI protocols can be an effective way to implement a complicated task rather than employing a complex BCI protocol. Although each simple protocol has limited bandwidth, it is enough to carry out a binary classification task. In addition, each simple protocol is trained relatively easily for such a simple task and easily operated. In this study, subjects required little training to adapt to each BCI protocol. Furthermore, its operation was relatively stable and robust, and could attain reliable accuracy for simple tasks. Their combination provided extended controllability. In a well-designed hybridization system, each simple protocol can sustain a sufficiently good accuracy per simple task mode within a complex task. Therefore, the overall performance of the composite task can be achieved with reliable accuracy. In addition, it was assessed that auxiliary signal processing techniques to the BCI modalities are also advantageous to enhance complicated task performance. In this work, a very simple image processing technique was useful to detect objects. As already pointed out in literature [9], [15]–[16], leveraging BCI with advanced intelligence techniques will enrich potential applications and increase practical feasibility.

This work also evaluated the feasibility of a low-cost recording system-based BCI implementation. The low-cost BCI headset may not provide precise measurements for advanced or complicated BCI protocols for complex tasks; however, it could still collect enough data to achieve reasonable performance with simple BCI protocols at simple tasks such as binary classification. This work also suggested and demonstrated that hybrid BCI facilitates the use of a low-cost EEG recording system and makes it possible to use it for complicated tasks by conducting real-time humanoid navigation/exploration and object recognition tasks.

The proposed hybrid scheme with the low-cost EEG headset attained average accuracies of 84.6%, 84.4%, and 89.5–91% for ERD, SSVEP, and P300 -based BCI protocols, depending on the number of objects respectively. Furthermore, it provides ITRs of up to 18.1 and 21.1 bits/min during navigation/exploration and recognition modes respectively. While switching between SSVEP and ERD, accuracy and bit rate tended to be lower. Its accuracy was 73% on average.

Our real-time humanoid robot navigation/exploration experiments showed that the proposed hybrid BCI scheme with a low-cost recording system achieved reasonably comparable performances to manual keyboard control. In addition, the proposed hybrid BCI control was comparable to asynchronous direct motor imagery-based BCI control [7] with respect to humanoid robot navigation.

Although the results demonstrate the possibility of applying a low-cost recording system to complicated tasks, some issues were recognized while conducting experiments. The electrode locations of the recording headset did not fully cover the sensorimotor cortex. It may be possible that the area not covered produces more effective brain activity for BCI protocol design. Additionally, due to the plastic connections between the electrodes and the headset's frame, the locations of the electrodes were subtly changed when the headset was worn. In the future, an in-house custom design of an EEG headset, whose number of electrodes and electrode locations are better optimized for specific tasks, will be attempted. In this work, the specific stimulus frequencies for SSVEP-based protocol were 12 and 15 Hz. The specifications of the low-cost headset limited the selection of the stimulus frequencies. If the frequencies were more clearly distinguished from the mu band for ERD-based protocol, a more robust classification between SSVEP and ERD performances would have been possible. Future work will develop a low-cost headset that can tune to higher stimulus frequencies more robustly.

## Conclusion

This paper addressed a low-cost hybrid BCI system-based approach for a humanoid robot navigation and object recognition. The robot movement and direction were commanded through the SSVEP and ERD-based BCIs and favorite object recognition was implemented by the P300 potential-based BCI with the help of a simple image processing technique. We believe this paper demonstrated the feasibility of carrying out multiple tasks through a combination of simple BCI protocols using a low-cost BCI system. This hybrid strategy has the potential to enable further applications using a low-cost BCI recording system, even though the quality or amount of data is lower than that of advanced BCI systems. In the future, we will continue to investigate the implementation of more practical BCI applications while making its usage friendlier to the user.

## Supporting Information

Video S1 Real-time humanoid navigation/exploration and recognition demo.
(MP4)
